# Conditional Ablation of PKCλ/ι in CD4^+^ T Cells Ameliorates Hepatic Fibrosis in *Schistosoma japonicum*-Infected Mice via T Follicular Helper (Tfh) Cell Suppression Coupled with Increased Follicular Regulatory T (Tfr) and Regulatory B (Breg) Cell Activities

**DOI:** 10.3390/biom15101430

**Published:** 2025-10-09

**Authors:** Congjin Mei, Yingying Yang, Panpan Dong, Julu Lu, Xinyue Zhang, Jingping Li, Lijun Song, Chuanxin Yu

**Affiliations:** National Health Commission Key Laboratory of Parasitic Disease Control and Prevention, Jiangsu Provincial Key Laboratory on Parasite and Vector Control Technology, Jiangsu Institute of Parasitic Diseases, Wuxi 214064, China; meicongjin@jipd.com (C.M.); yangyingying@jipd.com (Y.Y.); dongpanpan@jipd.com (P.D.); lujulu@jipd.com (J.L.); zhangxinyue@jipd.com (X.Z.); lijingping@jipd.com (J.L.)

**Keywords:** schistosomiasis japonica, hepatic fibrosis, *PKCλ/ι*, Tfh/Tfr, Breg, cellular interactions

## Abstract

To further investigate the role of *PKCλ/ι* in *Schistosoma japonicum*-induced hepatic fibrosis, we employed a CD4^+^ T-cell-specific *PKCλ/ι* conditional knockout (KOSJ) mouse model, with wild-type (WTSJ) mice used as controls. Transcriptomic profiling of hepatic mRNA was used to reveal the immune regulatory mechanisms underlying the role of *PKCλ/ι* in the hepatic fibrosis caused by *S. japonicum* infection. Flow cytometry, RT–qPCR and ELISA were used to analyze the effects of *PKCλ/ι* on Tfh and Tfr cells, and single-cell RNA sequencing was used to elucidate the interactions between Tfr and B cells. The results showed that *PKCλ/ι* deficiency led to altered BCR signaling gene expression, reduced germinal center activity, and decreased anti-SEA antibody levels. Tfh cells and key factors including *IL-21*, *CXCR5*, and *ICOS* were downregulated, while Tfr cells and IL-10^+^ B cells increased. Additionally, hepatic neutrophils decreased and Treg/Tfr ratios rose, with enhanced IL-10-mediated cellular crosstalk. These findings indicate that *PKCλ/ι* deficiency attenuates liver fibrosis by inhibiting Tfh differentiation, promoting Tfr function, and activating IL-10-producing Breg cells, suggesting its potential as a therapeutic target.

## 1. Introduction

Schistosomiasis remains one of the most prevalent parasitic diseases worldwide, affecting more than 250 million individuals globally [1]. Three primary species of schistosomes are pathogenic to humans: *Schistosoma japonicum* (*S. japonicum*), *Schistosoma mansoni*, and *Schistosoma haematobium* [2]. The pathogenesis of *S. japonicum* infection is predominantly caused by intrahepatic egg deposition. Egg-derived secreted/excreted products (ESPs) recruit inflammatory cells and activate hepatic stellate cells, initiating granulomatous inflammation and hepatic fibrosis around eggs through antigen-driven immune responses [3]. This pathological progression results in severe hepatic dysfunction and potentially fatal outcomes [4]. Therefore, elucidating the immunological mechanisms underlying *S. japonicum*-induced hepatic fibrosis not only provides a scientific foundation for developing targeted therapeutic and preventive strategies against schistosomiasis-associated liver fibrosis but also offers insights applicable to fibrotic liver pathologies of diverse etiologies, with significant implications for both biomedical research and public health.

A substantial body of evidence indicates that CD4^+^ T cell subsets, including Th1, Th2, Th17, Tfh (T follicular helper, Tfh), Th9, and Treg (regulatory T, Treg) cells, are critically involved in the pathogenesis of liver granuloma formation and fibrosis during schistosomiasis [5,6,7,8]. In the early stages of *S. japonicum* infection, the immune response is predominantly Th1-driven [8]. Following egg deposition, the response converts to a Th2-polarized state, characterized by the production of *IL-4*, *IL-13*, and TGF-β. These cytokines activate hepatic stellate cells, leading to an imbalance between extracellular matrix (ECM) synthesis and degradation, thereby promoting fibrosis around deposited eggs [9,10,11].

Emerging studies highlight the pivotal role of B cells in modulating schistosomal egg-induced granuloma formation and fibrosis, with evidence suggesting their regulatory influence on T-cell functionality [12,13]. Early-stage B-cell deficiency during infection impairs GC (germinal center, GC) development, reduces antibody production, and disrupts granuloma formation [14]. In addition to being involved in antibody secretion, B cells contribute to antigen presentation, costimulatory signaling, and cytokine production [15]. During T-B cell interactions, B-cell-derived co-stimulatory signals are essential for Th2 differentiation [16], whereas regulatory B cells (Bregs) suppress granulomatous inflammation via IL-10 secretion [17,18].

As a subset of CD4^+^ T cells, Tfh cells serve as critical facilitators of B-cell function. Tfh cells provide *IL-21* and *CD40L* signals, which are indispensable for both GC B-cell proliferation/differentiation and extrafollicular B-cell development [19]. Mature Tfh cells within GCs secrete IL-4 [20], driving effector B-cell differentiation and amplifying Th2-mediated inflammation [21]. Reciprocal signaling is necessary for maintaining the expression of the transcription factor *Bcl-6* in Tfh cells [22], and *ICOSL* promotes the interaction between Tfh and B cells [23]. Notably, Tfh cells are recruited to the liver during schistosome infection, where they express elevated levels of *ICOS* and *Bcl-6*, directly contributing to granuloma formation [24].

Tfr (T follicular regulatory, Tfr) cells, a recently identified CD4^+^ T subset, exhibit dual characteristics of Treg and Tfh cells. They express Foxp3 and secrete *IL-10* (the specific molecule of Tregs) while simultaneously displaying Tfh-associated markers such as PD-1, *CXCR5*, and *Bcl-6* [25,26]. *Bcl-6* expression is indispensable among many important factors for Tfr differentiation [27]. Tfr cells suppress the proliferation of Tfh and B cells in the GC in a CTLA-4 dependent manner to avoid the abnormal increase in Tfh cells that leads to autoimmune diseases [28]. Tfr cells can secrete anti-inflammatory cytokines such as *IL-10*, TGF-β, and granzyme B, which help regulate the intensity of the immune response and maintain immune homeostasis.

PKCλ/ι, an atypical protein kinase C (aPKC) isoform, regulates cellular metabolism [29,30], proliferation, and differentiation by phosphorylating serine/threonine residues. It plays critical roles in tumor immunity, allergic inflammation, and diabetes [31]. *PKCλ/ι* (human PKCι and murine PKCλ are orthologs exhibiting 98% amino acid sequence identity and are therefore collectively designated PKCλ/ι) is crucial for cell survival signal transduction [32] and has been suggested to be an essential isoform of protein kinase C for the development of multicellular organisms [33]. Previous studies have demonstrated that *PKCλ/ι* deficiency in CD4^+^ T cells suppresses Th2/Th17 differentiation, ameliorating schistosomiasis-induced hepatic fibrosis and house dust mite (HDM)-driven allergic asthma [34,35].

Further studies revealed that the deletion of *PKCλ/ι* inhibits the differentiation of CD4^+^ T cells into Th2 and Th17 cells but does not affect the differentiation of Th1 and Treg cells [34,35]. Compared with non-Tfh cell subsets (Th1, Th2, Th17, Th9, and Treg), Tfh cell subsets are the key helper cells for B cells to function. Tfh cells promote the proliferation of B lymphocytes through T-B interactions [23] or the secretion of IL-21 [36] and become plasma cells for antibody secretion, which plays an important role in the immune response and pathogenic mechanism [37]. Whether the absence of *PKCλ/ι* affects the differentiation and function of Tfh and Tfr cell subsets and subsequently affects their auxiliary effect on B cells and its role in liver lesions caused by schistosome infection have not yet been reported.

This study established a *S. japonicum* infection model using *PKCλ/ι* conditional gene knockout C57BL/6J mice to investigate the impact of *PKCλ/ι* deficiency on the differentiation of Tfh and Tfr cell, as well as their interplay with B cells. The results revealed that *PKCλ/ι* deletion in activated CD4^+^ T cells downregulated Tfh differentiation, enhanced Tfr and Breg functionality, and amplified the Tfr-mediated suppression of B cells. These changes reduced GC B-cell antibody secretion, highlighting *PKCλ/ι* as a key modulator in schistosomiasis-associated hepatic fibrogenesis.

## 2. Materials and Methods

### 2.1. Materials

#### 2.1.1. Mice

The *PKCλ/ι* conditional knockout mice (PKCλ/ι^flx/flx^Cre^OX40^ mice) were originally generated and maintained by the Model Animal Research Center of Nanjing University following Yang Junqi’s methods [34]. In our laboratory, these mice were bred, housed, and subjected to genotyping to screen for eligible *PKCλ/ι* conditional knockout mice for experimental use. All the animals were provided with free access to certified food and water and housed under specific pathogen-free (SPF) conditions. This study adhered to the Guidelines for the Care and Use of Laboratory Animals issued by the Ministry of Science and Technology of the People’s Republic of China (No. 398, 2006). All the experimental procedures were conducted in compliance with the General Requirements for Laboratory Biosafety in China (GB19489-2008) [38], and the ethical approval of Jiangsu Institute of Parasitic Diseases has been obtained (approval number: JIPD-2024-002.)

#### 2.1.2. Infectious Oncomelania Hupensis and Soluble Egg Antigen (SEA)

The infectious snails of the Jiangsu strain of *S. japonicum* were provided by the Oncomelania hupensis Laboratory, Jiangsu Institute of Parasitic Diseases. The Infected snails were immersed in dechlorinated water at 20 to 25 °C and exposed to an incandescent lamp for 2 h to hatch *S. japonicum* cercariae.

The SEA of *S. japonicum* was prepared and stored in our laboratory according to the literature [39].

### 2.2. Methods

#### 2.2.1. Establishment of *S. japonicum* Infection Model

Six- to eight-week-old wild-type C57BL/6J mice were randomly divided into two groups: wild-type mice without *S. japonicum* infection group (WT group, genotype PKCλ/ι^flx/flx^); wild-type mice with *S. japonicum*-infection group (WTSJ group, genotype PKCλ/ι^flx/flx^); and six- to eight-week-old *PKCλ/ι* conditional knockout mice were randomly divided into two groups: *PKCλ/ι* knockout mice without *S. japonicum* infection group (KO group, genotype PKCλ/ι^flx/flx^Cre^OX40^) and *PKCλ/ι* knockout mice with *S. japonicum* infection group (KOSJ group, genotype PKCλ/ι^flx/flx^Cre^OX40^). Each group consisted of 8 mice. For the infected groups, each mouse was percutaneously infected with 15 ± 1 *S. japonicum* cercariae via the abdominal patch method. At 7 weeks postinfection, the mice were anesthetized with CO_2_, and blood was collected via retro-orbital venous plexus puncture. The serum was separated and stored at −80 °C. Liver tissues were embedded and sectioned with hematoxylin and eosin (HE) and Masson’s trichrome staining using kits (Catalog No. G1120 and G1346, respectively) from Beijing Solarbio Science & Technology Co., Ltd (Beijing, China). HE and Masson-stained sections were taken pictures under an Olympus BX51 microscope (Tokyo, Japan). At least five images were captured from each mouse for analysis. The proportions of inflammatory areas and collagen deposition were quantified using Fiji software (windows-x64) with color deconvolution analysis [40,41]. For pathological HE analysis, specific steps are as follows: Using Fiji software (windows-x64) and the “Color Deconvolution” plugin, select “H&E” preset to separate inflammatory cells (light purple). Set a uniform threshold for the inflammatory cell channel images to ensure a consistent staining intensity judgment standard for all images. The software automatically calculates the pixel area of the inflammatory region in each field and the total pixel area of the entire field. For pathological Masson analysis, specific steps are as follows: Using the “Color Deconvolution” plugin, select the “Masson Trichrome” preset, separate the collagen fibers (blue) and muscle fibers (red). For the separated collagen channel images, set a uniform threshold to ensure a consistent staining intensity judgment standard for all images. The software automatically calculates the pixel area of the collagen positive region in each field and the total pixel area of the entire field. The collagen volume fraction is calculated according to the following formula, and the result is expressed as a percentage: CVF (%) = (pixel area of collagen positive region/total pixel area of the field) × 100%. Finally, the CVF value of each mouse is the average of all field CVF values.

#### 2.2.2. Detection of SEA-Specific IgG and IgG1 Antibodies in Mouse Serum

The microtiter plates were coated with SEA (1 µg/well in PBS) and incubated overnight at 4 °C, then blocked with 300 µL of 5% (*w*/*v*) non-fat dry milk in PBS per well for 1 h at 37 °C to prevent nonspecific binding; after three washes with PBST (PBS containing 0.05% Tween-20), 100 µL of diluted mouse serum (1:1000 in PBS) was added to each well and incubated for 1 h at 37 °C, followed by three PBST washes before adding either HRP-conjugated goat anti-mouse IgG (Proteintech, Wuhan, China, Cat# SA00001-1a; 1:1000 dilution) or IgG1 (Proteintech, Cat# SA00012-1; 1:1000 dilution) (100 µL/well) for 1 h at 37 °C; following three additional PBST washes, 100 µL of TMB substrate (Biolab, Ubi, Singapore, Cat# WE0309) was added per well and incubated for 10 min at room temperature in the dark before the reaction was terminated with 100 µL of 2 N H_2_SO_4_ and the absorbance at 450 nm was measured via a microplate reader (SpectraMax i3, Molecular Devices, San Jose, CA, USA).

#### 2.2.3. Flow Cytometry Analysis

Mouse spleens were aseptically collected and homogenized. After red blood cell lysis, the cell concentration was adjusted to 10 × 10^6^ cells/mL via RPMI 1640 medium. To maximize the detection signal for cytokines of T cell and B cell, the cells were stimulated with 2 µL/well of PMA (Leukocyte Activation Cocktail with BD GolgiPlug, BD Biosciences, San Jose, CA, USA, Cat# 550583) for 6 h before harvesting.

The stimulated cells were divided into three aliquots (2 × 10^6^ cells each) and subjected to sequential fixation and permeabilization with Fixation/Permeabilization solution (20 min, 4 °C) following the manual of the BD Cytofix/Cytoperm™ kit (BD Biosciences, San Jose, CA, USA, Cat# 554714) for subsequent cytokine staining in separate flow cytometry tubes:

To minimize nonspecific antibody binding, cells were incubated with an anti-mouse CD16/CD32 antibody for 10–15 min on ice for Fc receptor blocking prior to staining. The first cell aliquot was stained with the following fluorescently conjugated antibodies (1 μg each): PE-Cy7 rat anti-mouse CD4 (RM4-5; BD Biosciences, Cat# 552775), Brilliant Violet 650™ anti-mouse *CXCR5* (BioLegend, San Diego, CA, USA, Cat# 145517), BB700 mouse anti-mouse CD279 (PD-1; BD Biosciences, Cat# 566514), and IL-21-PE (Invitrogen, Waltham, MA, USA, Cat# 2622537) for the detection of CD4^+^PD-1^+^*CXCR5*^+^ Tfh cells and CD4^+^PD-1^+^IL-21^+^ cytokine-producing Tfh cells.

The second cell aliquot was stained with the following fluorescently conjugated antibodies (1 μg each):PE-Cy7 rat anti-mouse CD4 (RM4-5) (BD, Cat# 552775), CD25-APC (BioLegend, Cat# 102012), Bcl-6-BV421 (BD, Cat# 563363), and Foxp3-PE (BioLegend, Cat# 320008) for the detection of CD4^+^CD25^+^Foxp3^+^ Treg cells and CD4^+^Foxp3^+^Bcl-6^+^ Tfr cells.

The third cell aliquot was stained with the following fluorescently conjugated antibodies (1 μg each):PE/Cy7 anti-mouse IL-10 (BioLegend, Cat# 505026), CD95-BV650 (BioLegend, Cat# 152612), GL7-RB705 (BioLegend, Cat# 144610), CD19-APC (BioLegend, Cat# 115529), and IL-6-FITC (Invitrogen, Cat# 2281568) for the detection of CD19^+^IL-6^+^ effector B cells, CD19^+^IL-10^+^ regulatory B cells, and CD19^+^CD95^+^GL7^+^ GC B cells.

All groups (WT, WTSJ, KO, KOSJ) were processed under identical conditions, and a non-infection control (WT, KO group) was included to remove non-specific interference as much as possible. Tubes were incubated at 4 °C in the dark for 30 min. After two washes with PBS, the cells were resuspended in 400 μL of PBS and analyzed via a Beckman Coulter CytoFLEX S flow cytometer (San Jose, CA, USA), with triplicate measurements per sample. The detailed gating strategies were shown in Supplementary Figure S1. Data analysis was performed via CytExpert software (2.5.0.77).

#### 2.2.4. Real-Time Quantitative PCR (RT-PCR)

Liver tissues were collected from the mice in each group, and total RNA was extracted via the TRIzol method (Invitrogen, 15596026CN) following the manufacturer’s instructions. The First-strand cDNA was synthesized via reverse transcription via a reverse transcription kit (Roche, Basel, Switzerland) and served as the template for RT-PCR. RT-PCR was performed via SYBR Green I Master Mix (Roche, Basel, Switzerland) and the LightCycler 480 instrument (Roche, Basel, Switzerland) to detect the mRNA expression levels of BCR-associated factors (*LILRB4*, *FCGR2B*, *CD22*), Tfh cell-related factors (*Bcl-6*, *Blimp-1*, *IL-21*, *CXCR5*, *ICOS*), and NF-κB pathway components (*NF-κB1*) in mouse liver tissues. *18S* rRNA was used as the housekeeping gene for normalization of gene expression across all samples. The sequences of primer used are listed in Table 1.

#### 2.2.5. Western Blotting (WB)

Total liver protein was extracted with RIPA lysis buffer (1× PBS, 1% Nonidet P-40, 0.5% sodium deoxycholate, 0.1% SDS, 1 mM PMSF, supplemented with protease inhibitors). Equal protein aliquots (30 µg) were resolved by SDS-PAGE, transferred onto PVDF membranes, and immunoblotted with primary antibodies against P65 (Cell Signaling Technology, Danvers, MA, USA) and p-P65 (Cell Signaling Technology, Danvers, MA, USA). Protein bands were detected using a Cytiva Amersham ImageQuant800 system (Marlborough, MA, USA), using GAPDH as the loading control.

#### 2.2.6. Transcriptome Sequencing

Liver tissues from the same anatomical site in each group of mice were aseptically collected, immediately placed into cryovials, snap-frozen in liquid nitrogen for 30 min, and then transferred to a −80 °C freezer. The samples were subsequently transported on dry ice to Shanghai Meiji Biomedical Technology Co., Ltd (Shanghai, China). for eukaryotic transcriptome sequencing via the Illumina platform. Raw counts were analyzed with DESeq2 software (R4.1.2) (based on a negative binomial distribution) to identify differentially expressed genes (DEGs) between groups (screening criteria: *p*-adjust < 0.05 & |log2FC| ≥ 1). The relevant accession numbers are clearly provided in Supplementary Material part, named Supplementary Table S1 (RNA-Seq accession numbers).

#### 2.2.7. Single-Cell Sequencing

Liver tissues from three mice in the WTSJ group and three mice in the KOSJ group were pooled into one sample each. After being rinsed with PBS, the tissues were placed in tissue storage solution (Miltenyi, Bergisch Gladbach, Germany, 130-100-008) and transported via 4 °C cold chain to Shanghai Meiji Biomedical Technology Co., Ltd. for single-cell sequencing analysis. The 10× Genomics Chromium™ system (Pleasanton, CA, USA) was used for library preparation. Sequencing was performed on the NovaSeq Xplus/DNBSEQ-T7 high-throughput sequencing platform. The data were analyzed on the online platform of Majorbio Cloud Platform (www.majorbio.com). The raw reads were subjected to quality control via Fastp. The scRNA-seq data matrices was preprocessed by applying standard quality control filters based on three criteria: (1) UMI counts (number of unique molecular identifiers), (2) detected genes per cell, and (3) mitochondrial read percentage (using quartile-based threshold screening). The cell types were identified via marker genes via single R software (1.8.1). On this basis, cell to cell communication analysis (e.g., intercellular regulatory networks and receptor-ligand interactions) was performed using the R-based CellChat package (1.5.0). The relevant accession numbers are provided in Supplementary Material part, named Supplementary Table S2 (scRNA-Seq accession numbers).

#### 2.2.8. Statistical Analysis

Unless otherwise stated, the data in this study are presented as the means ± standard error of the mean (SEM). All the statistical analyses were performed via GraphPad Prism 9 (9.3.1.471). Statistical significance was assessed via Student’s t-test, one-way ANOVA, the Mann–Whitney U test, or the Kruskal–Wallis test, with *p* < 0.05 considered statistically significant.

## 3. Results

### 3.1. PKCλ/ι Deficiency Reduces Hepatic Granulomatous Pathology and Fibrosis in S. japonicum-Infected Mice

**Figure 1:**
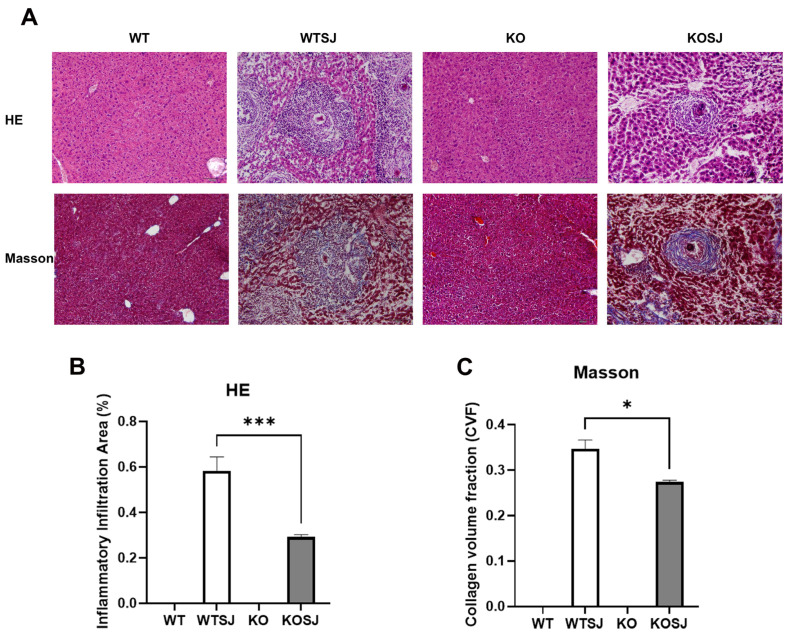
*PKCλ/ι* knockout alleviates the pathological changes in the liver of mice infected with *S. japonicum.* (**A**) Pathological HE staining & Collagen Masson staining of liver tissue sections in each group. (**B**) Statistical analysis results of the inflammatory infiltration area among different group. (**C**) Statistical analysis results of collagen volume fraction (CVF) among different each group. * *p* < 0.05, *** *p* < 0.001.

At seven weeks post-infection, infected wild-type control mice (WTSJ) exhibited pronounced liver egg granulomas and fibrosis (Figure 1A). The inflammatory infiltration area was significantly larger in WTSJ mice compared to PKCλ/ι-deficient mice (KOSJ) (0.58 ± 0.061 vs. 0.29 ± 0.011; *p* < 0.001; Figure 1B). Similarly, collagen volume fraction (CVF) was significantly higher in WTSJ controls (0.35 ± 0.019 vs. 0.27 ± 0.0043; *p* < 0.05; Figure 1C). These results demonstrate that *PKCλ/ι* loss significantly suppresses granuloma formation and attenuates fibrotic lesions.

### 3.2. Conditional PKCλ/ι Knockout Modulates B-Cell Functional Gene Expression in the BCR Signaling Pathway in S. japonicum-Infected Mice

**Figure 2:**
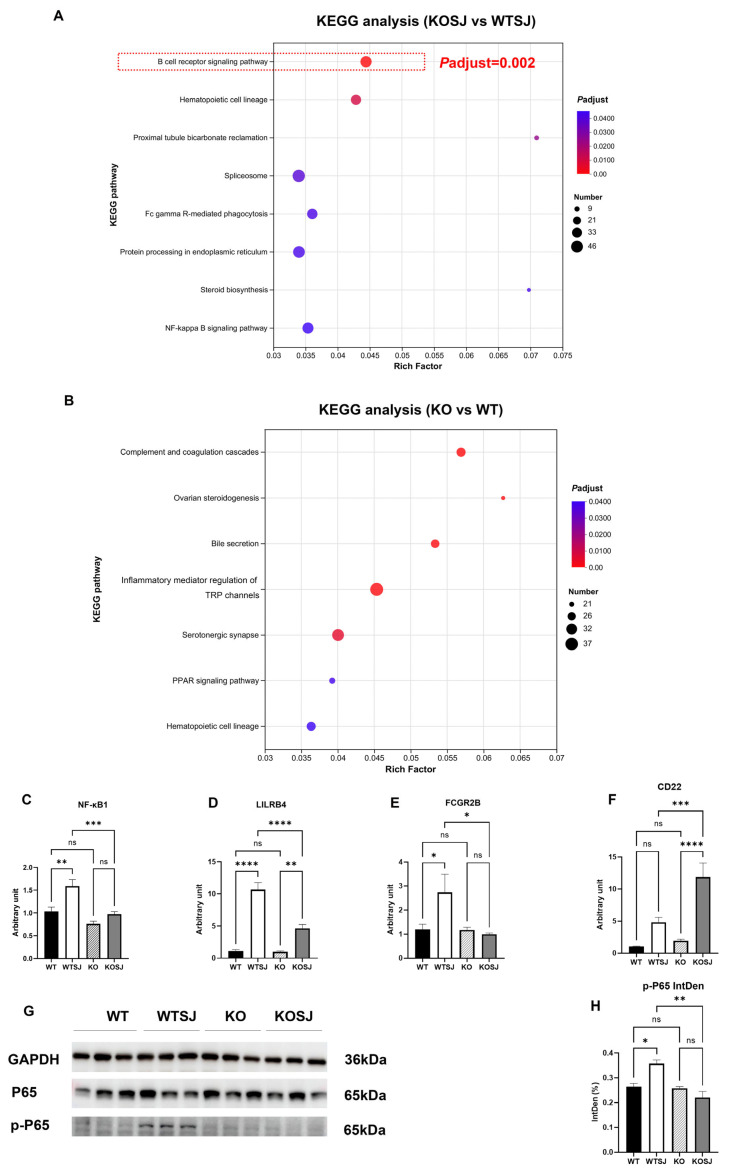
KEGG pathway enrichment analysis of differentially expressed genes in the liver transcriptome and PCR and WB validation. (**A**) KEGG pathways enriched with differentially expressed genes in the KOSJ group vs. the WTSJ group. (**B**) KEGG pathways enriched with differentially expressed genes in the KO group vs. the WT group. (**C**–**F**) RT–PCR validation of BCR pathway-related genes: *NF-κB1* (**C**), *LILRB4* (**D**), *FCGR2B* (**E**), and *CD22* (**F**). (**G**) Immunoblot of P65 and p-P65 expression in liver tissues. (**H**) Statistical analysis results of p-P65 expression. * *p* < 0.05, ** *p* < 0.01, *** *p* < 0.001, **** *p* < 0.0001; ns, not significant.

Comparative transcriptomic analysis and KEGG pathway enrichment of liver tissues across the four experimental groups revealed that the top eight enriched pathways for differentially expressed genes (DEGs) between the KOSJ and WTSJ groups included the B-cell receptor (BCR) signaling pathway, hematopoietic cell lineage, proximal tubule bicarbonate reclamation, spliceosome, Fc gamma R-mediated phagocytosis, protein processing in the endoplasmic reticulum, steroid biosynthesis, and the NF-κB signaling pathway. Strikingly, the BCR pathway demonstrated the most significant enrichment (adjusted *p* = 0.002; Rich Factor = 0.044, Figure 2A), with 39 DEGs annotated to this pathway. Notably, no significant alterations in BCR-related genes were detected between uninfected WT and KO groups (Figure 2B).

Transcriptomic profiling revealed dysregulation of critical BCR pathway genes: *NF-κB1* (proinflammatory regulator [42,43]) was downregulated; *LILRB4* (suppresses antigen-presenting cell (APC) and Tfh activity, indirectly attenuating B-cell antibody secretion [44]) was downregulated; *FCGR2B* (inhibits B-cell activation and antibody production via immune complex-mediated BCR- FCGR2B crosslinking [45]) was downregulated; and *CD22* (negative regulator of BCR signaling, limiting B-cell activation [46]) was markedly upregulated. Quantitative PCR validation of these genes in liver tissues yielded concordant results: *NF-κB1* (p50) expression levels (WT: 1.03 ± 0.093; WTSJ: 1.59 ± 0.14; KO: 0.76 ± 0.058; KOSJ: 0.97 ± 0.055) were significantly lower in KOSJ than in WTSJ (*p* < 0.001) (Figure 2C). *LILRB4* expression (WT: 1.12 ± 0.19; WTSJ: 10.65 ± 1.09; KO: 0.98 ± 0.14; KOSJ: 4.63 ± 0.62) was markedly lower in KOSJ than in WTSJ (*p* < 0.0001) (Figure 2D). *FCGR2B* expression (WT: 1.19 ± 0.22; WTSJ: 2.73 ± 0.76; KO: 1.17 ± 0.12; KOSJ: 0.99 ± 0.056) was significantly lower in KOSJ than in WTSJ (*p* < 0.05) (Figure 2E). CD22 expression (WT: 1.02 ± 0.080; WTSJ: 4.83 ± 0.76; KO: 1.93 ± 0.25; KOSJ: 11.88 ± 2.20; Figure 2F) was substantially greater in KOSJ than in WTSJ (*p* < 0.001). Additionally, the band intensities of p-P65 were quantified, yielding the following normalized gray values: 0.26 ± 0.013 (WT), 0.36 ± 0.015 (WTSJ), 0.26 ± 0.0062 (KO), and 0.22 ± 0.025 (KOSJ) (Figure 2H). Immunoblot analysis revealed decreased expression of p-P65 in KOSJ liver tissues compared to WTSJ controls (Figure 2G,H).

Collectively, these data demonstrate that conditional *PKCλ/ι* knockout suppresses the expression of proinflammatory (NF-κB1) and antibody secretion-associated (LILRB4, FCGR2B) genes within the BCR pathway while increasing the expression of the negative feedback regulator CD22 in *S. japonicum*-infected mice (Figure 2C–F).

### 3.3. Conditional PKCλ/ι Knockout Impairs B-Cell Function

**Figure 3:**
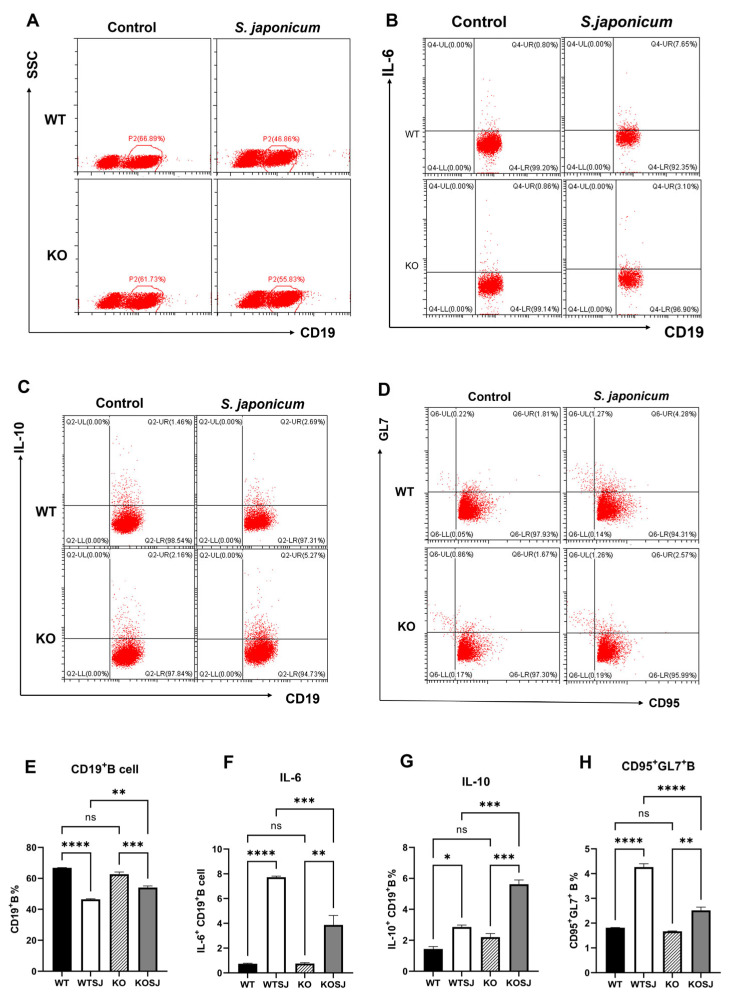
Effects of *PKCλ/ι* conditional knockout in activated T cells on B-cell quantity and function. (**A**) Flow cytometry dot plots showing total CD19^+^ B cells in the splenocyte population. (**B**) Flow cytometry dot plots of CD19^+^IL-6^+^ B cells in the splenocyte population. (**C**) Flow cytometry dot plots of CD19^+^IL-10^+^ B cells in the splenocyte population. (**D**) Flow cytometry dot plots showing the percentage of germinal center CD95^+^GL7^+^ B cells. (**E**) Statistical results of total CD19^+^ B-cell counts. (**F**) Statistical analysis of the proportion of CD19^+^IL-6^+^ B cells. (**G**) Statistical analysis of the proportion of CD19^+^IL-10^+^ B cells. (**H**) Statistical results of the proportion of CD95^+^GL7^+^ B cells in the germinal center. * *p* < 0.05, ** *p* < 0.01, *** *p* < 0.001, **** *p* < 0.0001; ns, not significant.

Splenocytes from each group were stimulated with PMA for 6 h, and 2 × 10^6^ cells were fixed, permeabilized, and analyzed by flow cytometry to assess the proportion of CD19^+^ B cells and the expression levels of IL-10/IL-6 intracellularly. The results revealed that after PMA stimulation the percentages of CD19^+^ B cells were 66.81 ± 0.12%, 46.41 ± 0.42%, 62.61 ± 0.95%, and 54.00 ± 1.10% in the WT, WTSJ, KO, and KOSJ groups, respectively. No significant difference was detected between uninfected WT and KO mice (*p* > 0.05). Compared with noninfected mice, *S. japonicum* infection markedly reduced B-cell numbers in the WTSJ group (*p* < 0.0001) and KOSJ group (*p* < 0.001), but KOSJ mice presented higher CD19^+^ B-cell counts than did WTSJ mice postinfection (*p* < 0.01) (Figure 3A,E). The decline in CD19^+^ B cells in the KOSJ group of mice was significantly lower than that in the WTSJ group.

After PMA stimulation, the percentages of IL-10^+^ CD19^+^ B cells were 1.44 ± 0.16%, 2.86 ± 0.12%, 2.20 ± 0.24%, and 5.62 ± 0.28% in the WT, WTSJ, KO, and KOSJ groups, respectively. Compared with WTSJ mice, KOSJ mice presented a significant increase in the proportion of IL-10-producing B cells (*p* < 0.001) (Figure 3C,G). The percentages of IL-6^+^ CD19^+^ B cells were 0.74 ± 0.042%, 7.73 ± 0.08%, 0.76 ± 0.075%, and 3.87 ± 0.77% in the WT, WTSJ, KO, and KOSJ groups, respectively. IL-6^+^ B cells were significantly lower in the KOSJ group than in the WTSJ group (*p* < 0.001) (Figure 3B,F). Moreover, the average proportions of GC CD95^+^GL7^+^ B cells in the WT, WTSJ, KO and KOSJ groups were 7.91 ± 0.32%, 14.28 ± 0.43%, 8.20 ± 0.18%, and 11.35 ± 0.28%, respectively. Compared with WTSJ mice, KOSJ mice presented a lower proportion of CD95^+^GL7^+^ B cells (*p* < 0.001) (Figure 3D,H).

**Figure 4:**
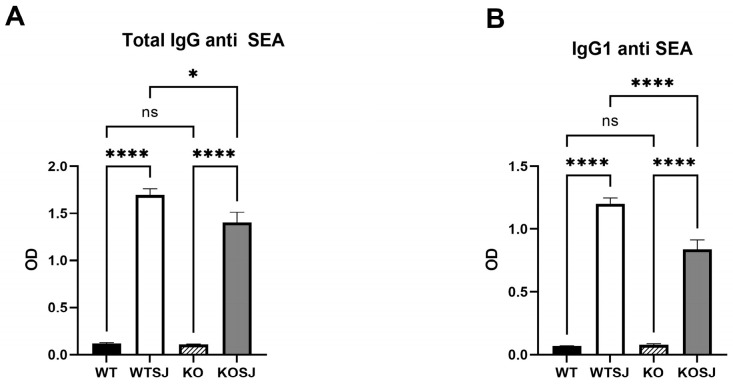
Effect of *PKCλ/ι* conditional knockout in activated T cells on IgG antibody production by B cells. (**A**) Serum levels of SEA-specific total IgG antibodies. (**B**) Serum levels of SEA-specific IgG1 antibodies. Data represent the mean ± SE from three independent experiments. * *p* < 0.05, **** *p* < 0.0001; ns, not significant.

ELISA analysis of the serum anti-SEA IgG levels revealed that the total anti-SEA IgG (OD450 nm) in the WT, WTSJ, KO, and KOSJ groups were 0.12 ± 0.0093, 1.70 ± 0.064, 0.11 ± 0.0038, and 1.40 ± 0.11, respectively. KOSJ mice presented significantly lower IgG levels than WTSJ mice did (*p* < 0.05) (Figure 4A). The serum levels of anti-SEA IgG1 (OD450 nm) in the WT, WTSJ, KO, and KOSJ groups were 0.07 ± 0.0010, 1.20 ± 0.046, 0.079 ± 0.0067, and 0.84 ± 0.078, respectively. The IgG1 levels were markedly lower in the KOSJ group than in the WTSJ group (*p* < 0.0001) (Figure 4B).

These findings indicate that *S. japonicum* infection decreases the proportion of splenic B cells, which is consistent with prior reports [12]. While conditional *PKCλ/ι* knockout did not affect B-cell counts in uninfected mice, it attenuated infection-induced B-cell depletion in KOSJ mice. Notably, *PKCλ/ι* deficiency increased the proportion of IL-10-producing regulatory B cells but reduced the proportion of IL-6-secreting effector B cells and antibody-producing GC (CD95^+^GL7^+^) B cells.

### 3.4. Conditional PKCλ/ι Knockout Downregulates Tfh Cell Differentiation in the Spleen and IL-21 Expression in S. japonicum-Infected Mice

**Figure 5:**
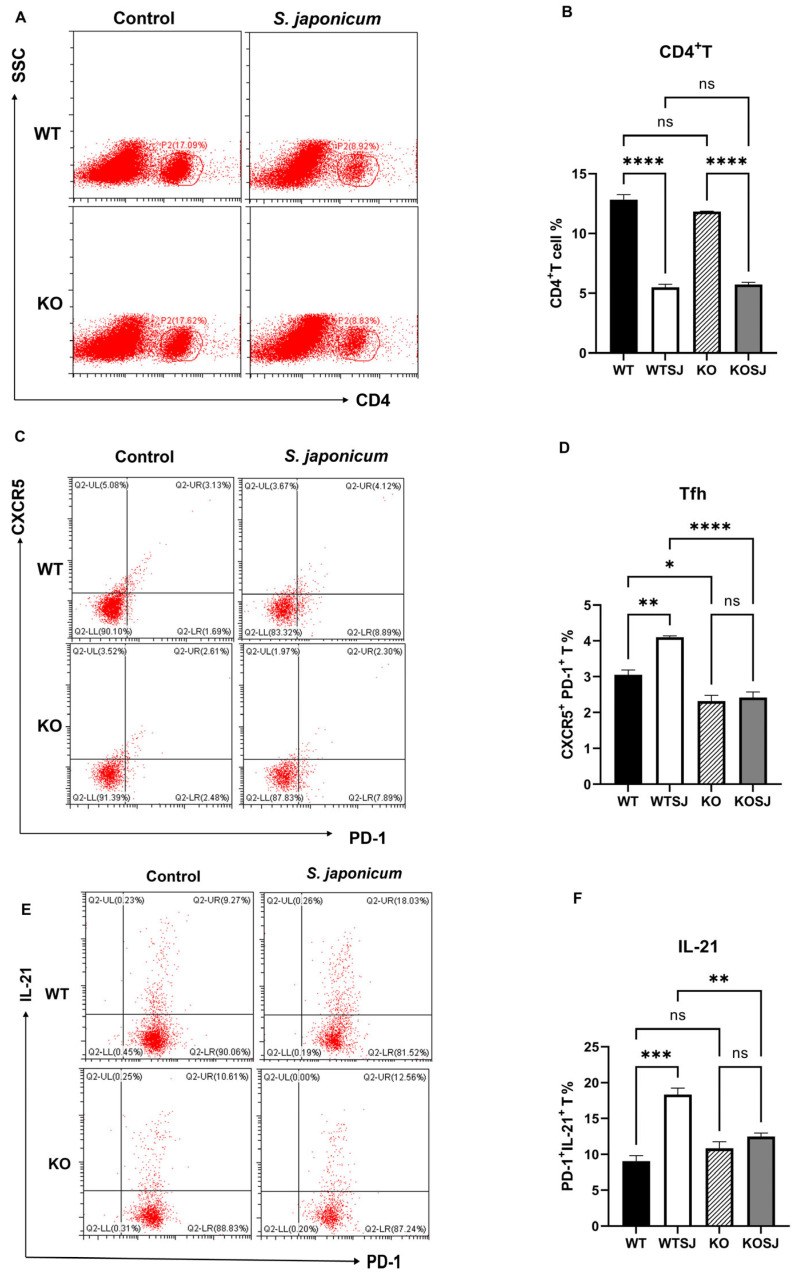
Effect of *PKCλ/ι* conditional knockout on splenic Tfh cell differentiation and function. (**A**) Flow cytometric analysis of CD4^+^ T-cell populations in splenocytes after 6 h of PMA stimulation. (**B**) Quantitative analysis of CD4^+^ T-cell proportions. (**C**) Flow cytometric assessment of Tfh cell (*CXCR5*^+^PD-1^+^) frequencies following 6 h of PMA stimulation. (**D**) Statistical analysis of Tfh cell proportions. (**E**) Flow cytometric evaluation of IL-21-producing cell frequencies after 6 h of PMA stimulation. (**F**) Quantitative analysis of CD4^+^PD-1^+^IL-21^+^ T-cell populations. * *p* < 0.05, ** *p* < 0.01, *** *p* < 0.001, **** *p* < 0.0001; ns, not significant.

Splenocytes were stimulated with PMA for 6 h, followed by fixation, permeabilization, and flow cytometry analysis of 2 × 10^6^ cells per group to assess the percentage of CD4^+^ T cells and Tfh subsets (*CXCR5*^+^PD-1^+^) within the spleen. After PMA stimulation, the results revealed that the percentages of CD4^+^ T cells in the WT, WTSJ, KO, and KOSJ groups were 16.64 ± 0.46%, 8.94 ± 0.12%, 17.51 ± 0.18%, and 8.93 ± 0.15%, respectively (Figure 5A,B). *S. japonicum* infection significantly reduced the proportion of CD4^+^ T cells in both the WTSJ and KOSJ groups compared with the uninfected controls (*p* < 0.0001), with no difference between infected KOSJ and WTSJ mice (*p* > 0.05). The proportions of *CXCR5*^+^PD-1^+^ Tfh cells among CD4^+^ T cells were 3.05 ± 0.14% in WT, 4.10 ± 0.042% in WTSJ, 2.32 ± 0.16% in KO, and 2.41 ± 0.16% in KOSJ after PMA stimulation. Compared with that in WT mice, the proportion of Tfh cells in infected WTSJ mice was greater (*p* < 0.01), but the proportion of Tfh cells in KOSJ mice was not significantly different from that in uninfected KO controls (*p* > 0.05). Notably, compared with the WTSJ group, the KOSJ group presented a significantly reduced proportion of Tfh cells (*p* < 0.0001) (Figure 5C,D). The proportions of PD-1^+^IL-21^+^ cells were as follows: WT (9.05 ± 0.78%), WTSJ (18.33 ± 0.91%), KO (10.83 ± 0.93%), and KOSJ (12.47 ± 0.49%) (Figure 5E,F). KOSJ mice presented markedly fewer PD-1^+^IL-21^+^ cells than WTSJ mice did (*p* < 0.01), with no difference compared with uninfected KO mice (*p* > 0.05).

These data demonstrate that conditional *PKCλ/ι* knockout does not alter total splenic CD4^+^ T-cell proportion but rather suppresses infection-driven Tfh cell differentiation and attenuates *IL-21* upregulation in Tfh cells during *S. japonicum* infection.

### 3.5. Conditional PKCλ/ι Knockout Enhances Tfr Cell Differentiation Post-S. japonicum Infection

**Figure 6:**
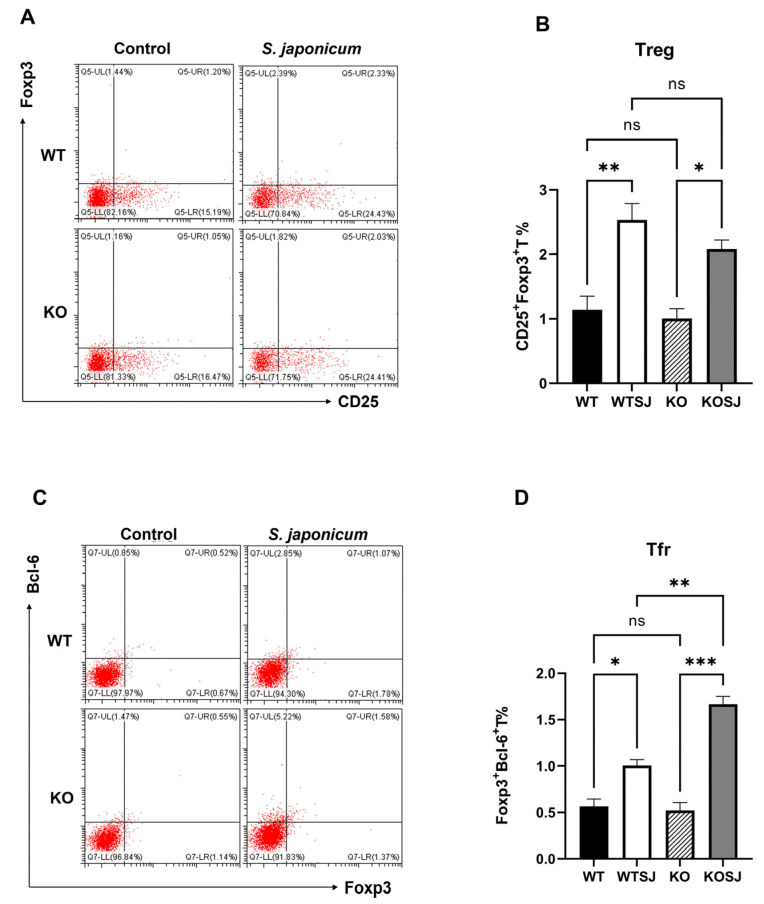
Effects of *PKCλ/ι* conditional knockout in activated T cells on splenic Tfr cell proportions and Bcl-6 expression. (**A**) Flow cytometric analysis of Treg (Foxp3^+^) cell frequencies in splenocytes after 6 h of PMA stimulation. (**C**) Flow cytometric assessment of Tfr (*CXCR5*^+^Foxp3^+^) cell frequencies following 6 h of PMA stimulation. (**B**) Statistical analysis of Treg proportions. (**D**) Quantitative evaluation of Tfr cell frequencies. * *p* < 0.05, ** *p* < 0.01, *** *p* < 0.001, ns, not significant.

Splenocytes were stimulated with PMA for 6 h, and 1 × 10^6^ cells per group were fixed, permeabilized, and analyzed via flow cytometry. The results revealed that the average proportions of CD4^+^CD25^+^Foxp3^+^ Treg cells were 1.14 ± 0.21% in WT, 2.53 ± 0.25% in WTSJ, 1.00 ± 0.15% in KO, and 2.08 ± 0.14% in KOSJ. Compared with the uninfected controls, both the WTSJ and KOSJ groups presented significant increases in Treg cells (*p* < 0.05), with no difference between the infected groups (*p* > 0.05) (Figure 6A,B). The proportions of Bcl-6^+^Foxp3^+^ Tfr cells were as follows: WT (0.58 ± 0.084%), WTSJ (1.00 ± 0.065%), KO (0.52 ± 0.08%), and KOSJ (1.66 ± 0.08%). Compared with WTSJ mice, KOSJ mice presented a significantly greater proportion of Tfr cells (*p* < 0.01) (Figure 6C,D).

These findings indicate that conditional *PKCλ/ι* knockout does not alter infection-induced splenic Treg expansion but markedly enhances follicular regulatory T (Tfr) cell differentiation during *S. japonicum* infection.

### 3.6. Conditional PKCλ/ι Knockdown Suppresses Hepatic Expression of Tfh Cell Differentiation-Associated Factors

**Figure 7:**
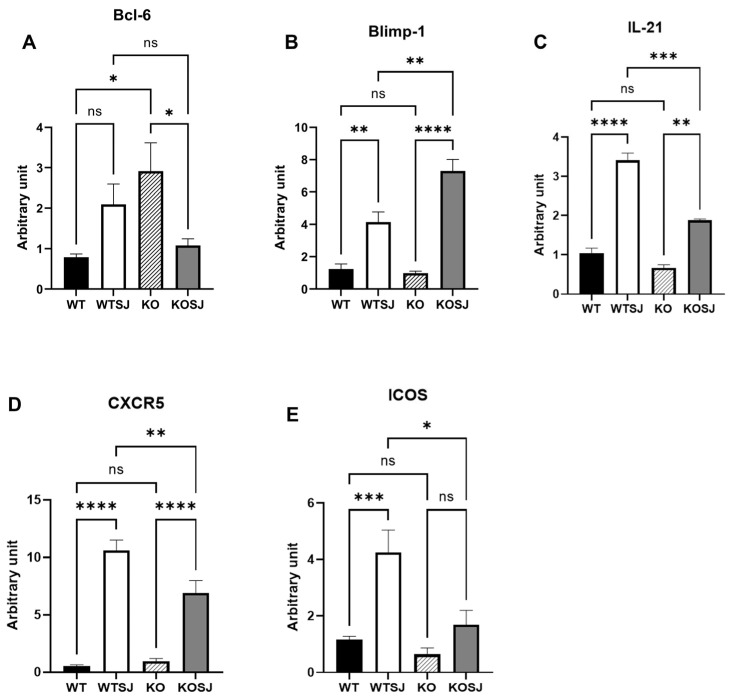
*PKCλ/ι* conditional knockout in activated T cells downregulates the mRNA expression of Tfh-related factors. (**A**) Hepatic Bcl-6 mRNA expression levels. (**B**) Hepatic Blimp-1 mRNA expression levels. (**C**) Hepatic IL-21 mRNA expression levels. (**D**) Hepatic *CXCR5* mRNA expression levels. (**E**) Hepatic *ICOS* mRNA expression levels. Data represent the mean ± SE from three independent experiments. * *p* < 0.05, ** *p* < 0.01, *** *p* < 0.001, **** *p* < 0.0001; ns, not significant.

The hepatic mRNA levels of Bcl-6 and Blimp-1 were quantified via RT–PCR, and the Bcl-6 expression levels in the liver tissue were as follows: WT (0.83 ± 0.072), WTSJ (2.01 ± 0.39), KO (4.60 ± 0.83), and KOSJ (1.69 ± 0.52). Compared with that in the WT group, the level of the Bcl-6 mRNA was significantly increased in the KO group (*p* < 0.0001). *S. japonicum* infection increased Bcl-6 levels in the WTSJ group (infected wild-type mice) compared with those in the uninfected WT control group (*p* < 0.05). Conversely, compared with uninfected KO mice, infected KOSJ mice presented a marked reduction in Bcl-6 expression (*p* < 0.001) (Figure 7A). The expression levels of Blimp-1 in liver tissue were as follows: WT (1.24 ± 0.30), WTSJ (4.14 ± 0.62), KO (0.98 ± 0.13), and KOSJ (7.30 ± 0.71) (Figure 7B). No difference was observed between WT and KO mice (*p* > 0.05). Infection significantly increased Blimp-1 in WTSJ (*p* < 0.01) and KOSJ (*p* < 0.001) versus uninfected controls, with KOSJ showing greater upregulation than WTSJ (*p* < 0.01). The hepatic mRNA levels of *IL-21*, *CXCR5*, and *ICOS* in the mice in each group were further assessed via RT–PCR, and the results revealed that the mRNA levels of *IL-21* were as follows: WT (1.05 ± 0.12), WTSJ (3.41 ± 0.18), KO (0.69 ± 0.077), and KOSJ (1.88 ± 0.035). KOSJ exhibited significantly lower *IL-21* levels than WTSJ did (*p* < 0.001) (Figure 7C). The mRNA levels of *CXCR5* were as follows: WT (1.08 ± 0.15), WTSJ (18.73 ± 2.47), KO (1.87 ± 0.49), and KOSJ (12.49 ± 2.28). *CXCR5* expression was markedly lower in KOSJ than in WTSJ (*p* < 0.01) (Figure 7D). The mRNA levels of *ICOS* were as follows: WT (1.17 ± 0.11), WTSJ (4.25 ± 0.79), KO (0.64 ± 0.22), and KOSJ (1.69 ± 0.52). *ICOS* expression was significantly lower in KOSJ than in WTSJ (*p* < 0.05) (Figure 7E).

These data demonstrate that CD4^+^ T-cell-specific *PKCλ/ι* knockout suppresses the hepatic expression of Tfh-associated factors (*Bcl-6*, *IL-21*, *CXCR5*, and *ICOS*) while increasing the expression of the inhibitory regulator Blimp-1 (which suppresses the expression of Bcl-6).

### 3.7. Single-Cell Sequencing Reveals the Mechanism by Which Conditional PKCλ/ι Gene Deletion Alleviates Hepatic Fibrosis

#### 3.7.1. *PKCλ/ι* Gene Deletion Alters the Composition of Immune Cell Types in the Liver

**Figure 8:**
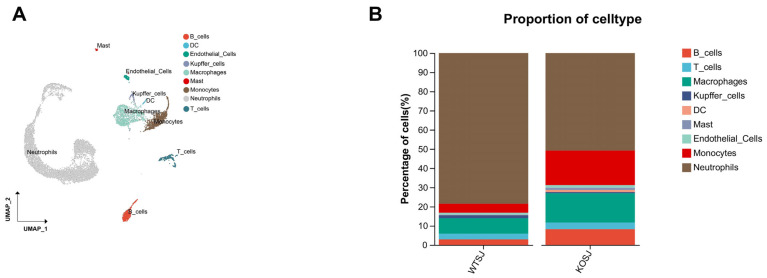
Single-cell RNA sequencing analysis of hepatic cell populations in activated T cells from mice with *PKCλ/ι* conditional knockout. (**A**) Scatter plot of cell types. (**B**) Cellular composition distribution in WTSJ group and KOSJ group.

After parenchymal cells were removed from a single-cell sample of two liver tissues, a total of 10,123 single cells were obtained. There were 5689 cells (56.20%) in the WTSJ group and 4431 cells (43.77%) in the KOSJ group. Cluster analysis revealed that these hepatic single cells aggregated into 20 clusters, which were annotated into 9 cell types on the basis of marker genes: B cells, T cells, macrophages, Kupffer cells, dendritic cells (DCs), endothelial cells, mast cells, monocytes, and neutrophils (Figure 8A). The proportions of immune cell subtypes were as follows: the proportions of B cells in the WTSJ group and the KOSJ group were 2.96% and 8.23%, respectively; the proportions of T cells in the WTSJ group and the KOSJ group were 2.87% and 3.45%, respectively. The proportions of macrophages in the WTSJ group and the KOSJ group were 8.14% and 15.28%, respectively. The proportions of Kupffer cells in the WTSJ group and the KOSJ group were 1.51% and 0.6%, respectively. The proportions of DCs in the WTSJ group and the KOSJ group were 0.09% and 1.11%, respectively. The proportions of mast cells in the WTSJ group and the KOSJ group were 0.19% and 1.16%, respectively. The proportions of endothelial cells in the WTSJ and KOSJ groups were 1.01% and 1.38%, respectively, and the proportions of monocytes in the WTSJ and KOSJ groups were 4.73% and 17.99%, respectively. The percentages of neutrophils in the WTSJ group and the KOSJ group were 78.51% and 50.8%, respectively (Figure 8B). Compared with those in wild-type mice infected with *S. japonicum* (WTSJ group), the proportions of B cells, T cells, macrophages, DCs, mast cells, endothelial cells and monocytes in the KOSJ group were significantly higher, but the proportion of neutrophils and Kupffer cells were significantly lower.

#### 3.7.2. *PKCλ/ι* Gene Deletion Increases the Tfr Cell Subpopulation in the Liver

**Figure 9:**
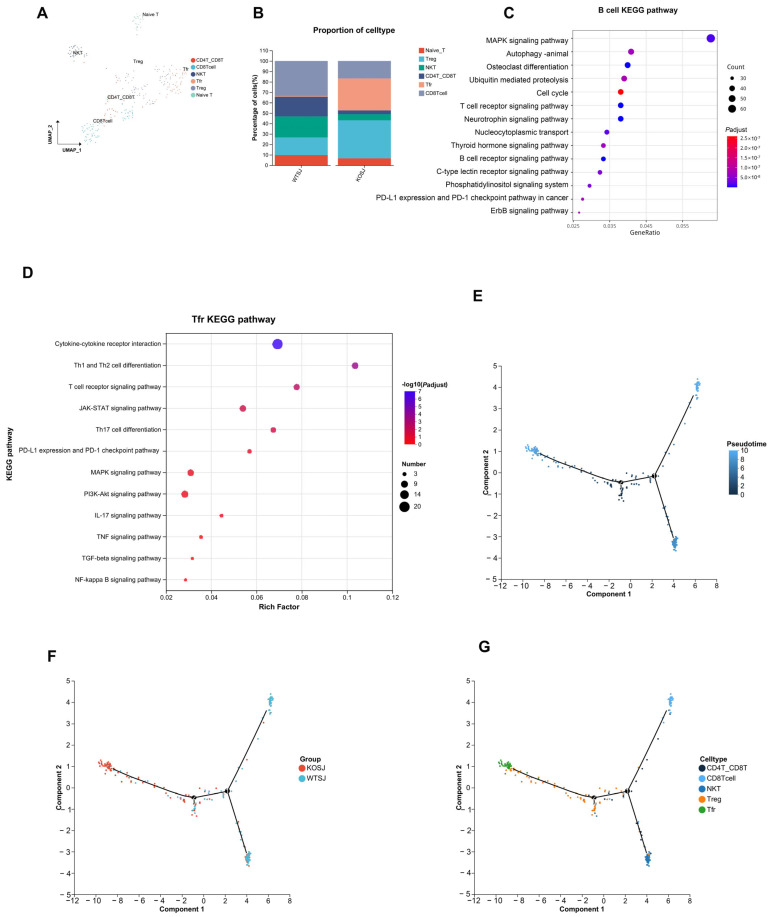
Single-cell RNA sequencing reveals altered T-cell subsets in the liver tissue of mice with T-cell-specific *PKCλ/ι* conditional knockout. (**A**) Scatter plot of T-cell subtypes. (**B**) Proportional distribution of T-cell subsets in WTSJ group and KOSJ group. (**C**) Bubble plot of KEGG pathway enrichment for differentially expressed genes in B cells versus other subsets. (**D**) Bubble plot of KEGG pathway enrichment for differentially expressed genes in Tfr cells versus other subsets. (**E**) Pseudotime trajectory analysis. (**F**) Cell differentiation trajectories across experimental groups. (**G**) Trajectory mapping of T-cell developmental stages.

T cells play a critical role in schistosome infection immunity. To comprehensively investigate T-cell functional characteristics, we performed clustering analysis on T cells. The results revealed that T cells aggregated into 10 clusters, which were annotated into 6 functional subtypes on the basis of marker genes: CD8^+^ T cells, CD4^+^CD8^+^ T cells, naïve T cells, Treg cells, Tfr cells, and NKT cells (Figure 9A). The proportions of CD8^+^ T cells in the WTSJ group and the KOSJ group were 33.57% and 16.79%, respectively. The proportions of CD4^+^CD8^+^ T cells in the WTSJ group and the KOSJ group were 18.88% and 3.65%, respectively. The proportions of naïve T cells in the WTSJ group and the KOSJ group were 9.79% and 6.57%, respectively. The proportions of Treg cells in the WTSJ group and the KOSJ group were 16.78% and 36.5%, respectively. The proportions of Tfr cells in the WTSJ group and the KOSJ group were 0.70% and 30.66%, respectively. The proportions of NKT cells in the WTSJ group and the KOSJ group were 20.28% and 5.84%, respectively (Figure 9B). *PKCλ/ι* knockout significantly increased the proportion of Tfr and Treg cells in murine liver tissue. Functional pathway analysis of differentially expressed genes through enrichment analysis revealed that Tfr cell-associated genes were enriched predominantly in KEGG pathways, including cytokine–cytokine receptor interaction, Th1/Th2 cell differentiation, the TCR signaling pathway, the JAK–STAT signaling pathway, and Th17 cell differentiation (Figure 9D). To delineate transcriptomic alterations in B cells, we performed differential expression analysis comparing KOSJ versus WTSJ using Seurat “https://cloud.r-project.org/web/packages/Seurat/index.html” (accessed on 16 April 2025). Applying stringent thresholds (|log_2_FC| > 0.3 and adjusted *p*-value < 0.5), we identified 2164 significantly differentially expressed genes (DEGs). KEGG enrichment of DEGs revealed MAPK signaling pathway (FDR = 0.063) and Autophagy (FDR = 0.041) (Figure 9C).

Pseudotime trajectory analysis demonstrated that hepatic T-cell developmental trajectories in WTSJ and KOSJ mice comprised 2 nodes and 5 distinct phases (Figure 9E). The differentiation trajectories revealed opposing developmental orientations between WTSJ and KOSJ T cells, indicating functional state divergence between groups (Figure 9F). Temporal evolution analysis of the cellular subtypes suggested that CD4^+^CD8^+^ T cells occupied earlier developmental stages than Tfr and Treg cells (Figure 9G).

These data demonstrate that conditional *PKCλ/ι* knockout in CD4^+^ T cells promotes the development of Tfr and Treg cells while delays the differentiation of pro-inflammatory related T-cell populations.

#### 3.7.3. *PKCλ/ι* Deletion Enhances Tfr-Breg Cell Interactions

**Figure 10:**
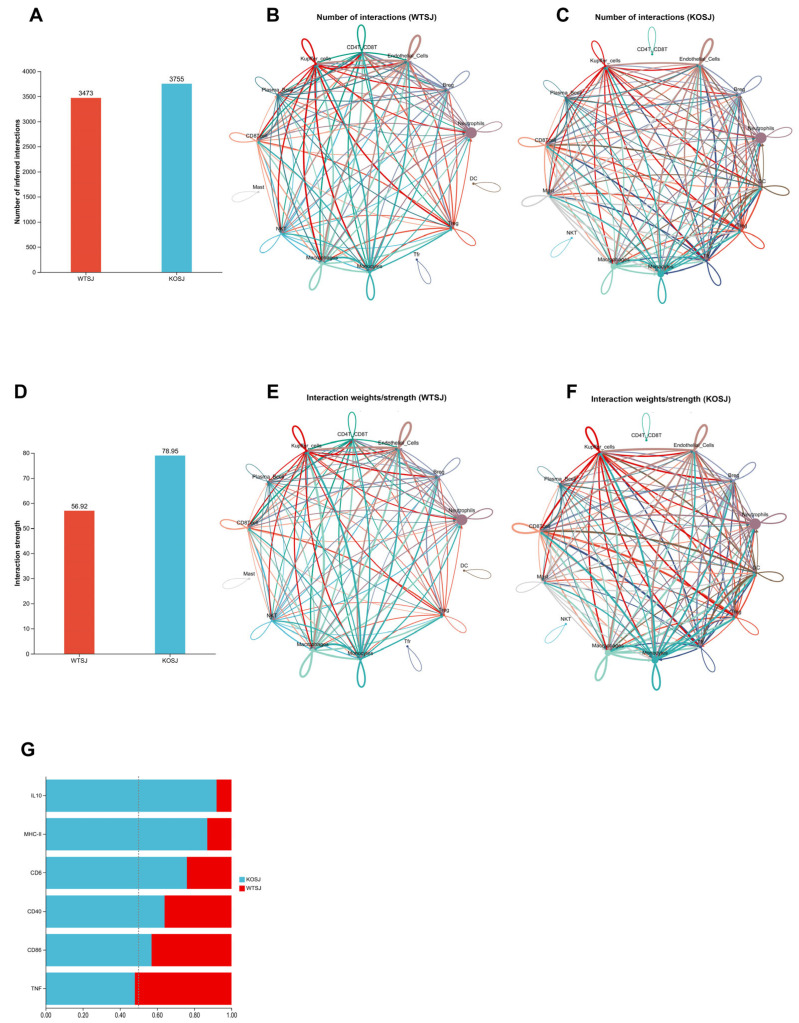
Alterations in cellular communication following *PKCλ/ι* knockout. Global changes in cell number (**A**) and interaction strength (**D**). (**B**) Number of cell–cell interactions in the WTSJ group. (**E**) Strength of cell–cell interactions in the WTSJ group. (**C**) Number of cell–cell interactions in the KOSJ group. (**F**) Strength of cell–cell interactions in the KOSJ group. (**G**) Differences in intercellular communication between WTSJ and KOSJ group. Legend: Solid circles of different colors represent distinct cell populations; arrow-originating nodes indicate ligand-producing cells; arrowhead-pointing nodes indicate receptor-expressing cells; interaction quantity network: node size corresponds to cell population abundance; edge thickness reflects the number of detected interactions; edge color matches the ligand-producing cell type; interaction strength network: edge thickness represents interaction intensity; edge color corresponds to the ligand-producing cell type.

The liver tissues of the WTSJ and KOSJ groups exhibited 3473 and 3755 cellular interactions, with interaction strengths of 56.92% and 78.95%, respectively (Figure 10A,D), indicating increased amounts of cellular interaction and intensity in the KOSJ group. Compared with those in the KOSJ group, the proportion (Figure 10B) and intensity (Figure 10E) of interactions between Tfr cells and Breg cells in the WTSJ group were lower. Global signaling pathway analysis revealed that *PKCλ/ι* knockout enhanced anti-inflammatory/immunoregulatory pathways such as the IL-10 signaling pathway and T-cell activation-related pathways (MHC-II, CD40, CD86, and CD6 signaling) but suppressed proinflammatory pathways such as the TNF signaling pathway (Figure 10G).

#### 3.7.4. *PKCλ/ι* Deletion Potentiates IL-10 Signaling Pathway

**Figure 11:**
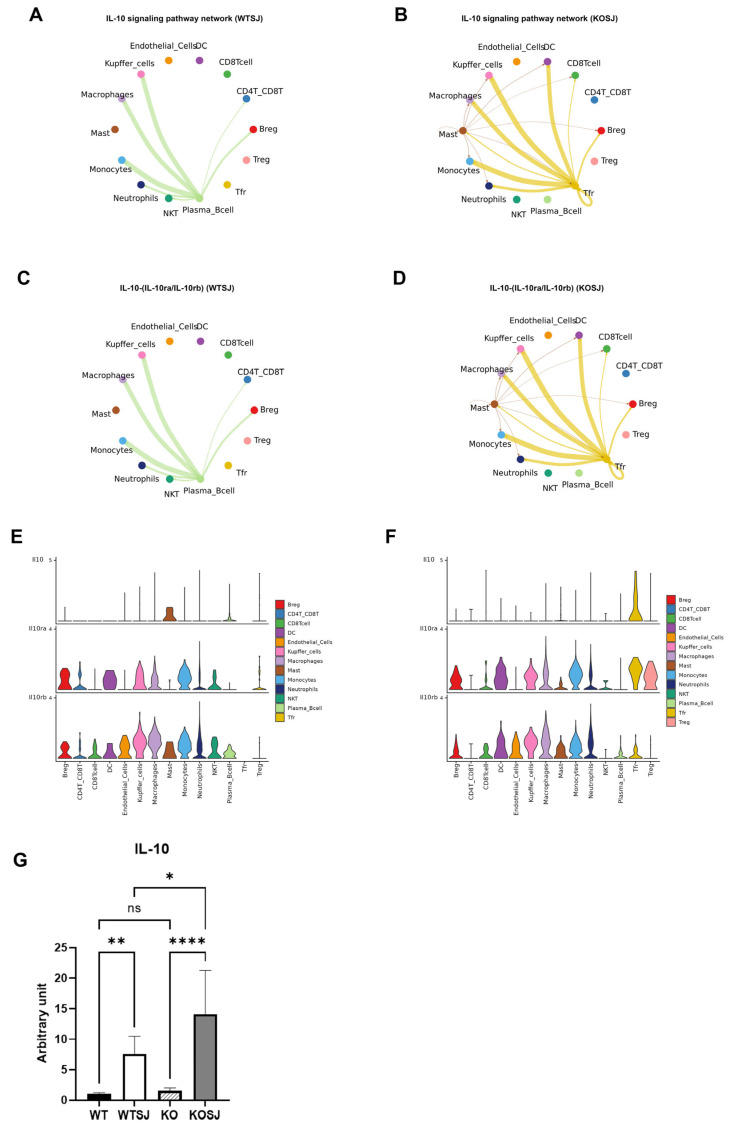
Intercellular communication networks of signaling pathways following *PKCλ/ι* conditional knockout. (**A**) IL-10 signaling pathway interactions among cell subsets in the WTSJ group. (**B**) IL-10 signaling pathway interactions among cell subsets in the KOSJ group. (**C**) IL-10-(IL-10ra/IL-10rb) ligand–receptor pairs across cell types in the WTSJ group. (**D**) IL-10-(IL-10ra/IL-10rb) ligand–receptor pairs across cell subsets in the KOSJ group. (**E**) Expression levels of IL-10-(IL-10ra/IL-10rb) components in WTSJ cell populations. (**F**) Expression levels of IL-10-IL-10ra/IL-10rb components in KOSJ cell populations. (**G**) mRNA expression quantification of IL-10 in liver tissues. Note: X-axis: Cell types; Y-axis: Normalized expression values of ligand/receptor genes in the pathway. Data represent the mean ± SE from three independent experiments. * *p* < 0.05, ** *p* < 0.01, **** *p* < 0.0001; ns, not significant.

Ligand–receptor analysis revealed predominant activation of the IL-10 signaling pathway following *PKCλ/ι* knockout (Figure 10G). In the WTSJ group, the IL-10 signaling pathway involved mainly interactions between various cells and plasma cells, but in the KOSJ group, the IL-10 signaling pathway involved mainly interactions between each cell and Tfr (Figure 11A,B). The results of the IL-10 receptor–ligand dynamics analysis revealed that the IL-10-IL-10ra/IL-10rb pairs occurred mainly between plasma cells and other cell types in the WTSJ group (Figure 11C), but this interaction shifted to Tfr and various cells in the KOSJ group (Figure 11D). Cellular communication network analysis of ligand–receptor gene pairs in the pathway revealed distinct IL-10 expression patterns. The anti-inflammatory cytokine IL-10 was predominantly expressed by mast cells in the WTSJ group (Figure 11E), but its expression was elevated in Tfr cells in the KOSJ group (Figure 11F). This shift in the cellular source was further validated via violin plot analysis. The RT–PCR results revealed that the mRNA level of IL-10 in heptic tissues across groups was as follows: WT, 1.03 ± 0.076; WTSJ, 7.60 ± 1.01; KO, 1.56 ± 0.17; and KOSJ, 14.10 ± 2.71. The significant elevation of IL-10 in KOSJ versus WTSJ (*p* < 0.05, Figure 11G) corroborated the single-cell sequencing findings, further confirming enhanced IL-10 production upon *PKCλ/ι* deletion.

## 4. Discussion

*S. japonicum* egg-induced hepatic granulomatous inflammation and fibrotic lesions constitute critical pathological features in schistosomiasis japonica. Emerging data have demonstrated that during the chronic phase of *S. japonicum* infection, Th2-dominant immune polarization mediated by egg antigens plays a pivotal role in modulating granulomatous inflammation and fibrogenesis. The Th2-derived cytokines IL-4 and IL-13 [47,48] activate hepatic stellate cells, driving excessive extracellular matrix (ECM) deposition—a hallmark of hepatic fibrosis. In addition to T cells, emerging evidence highlights the involvement of B cells in schistosomal granuloma formation. B-cell depletion impairs early granuloma development in infected mice [14,49], whereas IL-10-producing regulatory B1 cells attenuate hepatic pathology [50].

The pathological results of this study showed that *PKCλ/ι* knockout effectively alleviated the inflammatory and fibrosis of the egg granuloma (Figure 1). Our previous work revealed that conditional *PKCλ/ι* knockout in CD4^+^ T cells alleviates *S. japonicum*-induced hepatic granulomatous inflammation and fibrosis by suppressing Th2 polarization and IL-4^+^ CD4^+^ T-cell expansion [48].

In this study, transcriptomic analysis of hepatic tissues from *S. japonicum*-infected mice revealed distinct molecular signatures between the wild-type (WTSJ) and CD4^+^ T-cell-specific *PKCλ/ι* knockout (KOSJ) groups. Downregulation of NF-κB pathway components downstream of B-cell receptor activation, reduced expression of antibody production-associated genes (LILRB4, FCGR2B), and significant upregulation of CD22, a key negative regulator of B-cell activation, were found in CD4^+^ T-cell-specific *PKCλ/ι* knockout (KOSJ) group mice post-infection. The transcriptomic findings were also validated through hepatic mRNA level assays via RT–PCR (Figure 2C–F). The WB experiment confirmed that the phosphorylation level of the key protein P65 in the NF-κB pathway of the liver was decreased after *PKCλ/ι* was knocked out (Figure 2G,H). These findings suggest that conditional deletion of the *PKCλ/ι* gene in CD4^+^ T cells downregulates B-cell function.

Further monitoring of the levels of total IgG and IgG1-specific antibodies against SEA in the serum of mice infected with *S. japonicum* via ELISA revealed that the levels of total IgG and IgG1 antibodies against SEA in the sera of KOSJ mice were significantly lower than those in the sera of WTSJ mice (Figure 4A,B). The results of flow cytometry analysis revealed that conditional deletion of *PKCλ/ι* gene had no influence on the total proportion of B cells in the spleens of uninfected mice and that *S. japonicum* infection decreased the proportion of B cells in the spleens of wild-type and CD4^+^ T-cell-specific *PKCλ/ι* knockout mice [12]. However, the decrease in the proportion of B cells in the spleens with conditional deletion of the *PKCλ/ι* was relatively small. The proportion of B cells in spleens of the mice with gene deletion was greater than that of the wild-type mice with schistosomiasis, and the difference was statistically significant (Figure 3A,E).

The proportion of CD95^+^GL7^+^ B cells in the spleens of schistosome-infected *PKCλ/ι* knockout mice was significantly lower than that in the spleens of infected wild-type mice (Figure 3D,H). The proportion of effector B cells that secreted IL-6 was also markedly lower than that in wild-type mice (Figure 3B,F), whereas the proportion of Bregs that secreted IL-10 was significantly greater (Figure 3C,G). These data indicate that conditional knockout of the *PKCλ/ι* gene in CD4^+^ T cells not only downregulates the schistosome infection-induced Th2-type immune response [48] but also suppresses effector B-cell function while enhancing Breg activity. Previous reports suggest that in schistosomiasis, B10 cells may mitigate egg-induced liver tissue damage and fibrosis through the secretion of IL-10 [51]. Collectively, these results demonstrate that conditional *PKCλ/ι* deficiency in CD4^+^ T cells inhibits B-cell function and suppresses B-cell-mediated hepatic egg granulomatous inflammation in schistosome-infected mice by promoting the expansion of IL-10-secreting Bregs and downregulating the function of IL-6-secreting effector B cells. Given that Bregs can promote Treg differentiation, this might be the reason for the significant increase in Treg levels in the livers of *PKCλ/ι* deficiency mice [52].

Studies have shown that B-cell function is highly dependent on T-cell assistance, with Tfh cells playing a critical role in liver fibrosis lesions caused by schistosomiasis. Naïve CD4^+^ T cells differentiate into either Tfh cells (Bcl-6^+^CD4^+^ T cells) or non-Tfh cells (Blimp-1^+^CD4^+^ T cells) under dendritic cell (DC) regulation [22,53,54,55]. This bifurcation is driven by the mutually antagonistic transcription factors Bcl-6 and Blimp-1 [56], with Blimp-1 inhibiting CD4^+^ T-cell differentiation toward Bcl-6^+^ Tfh cells. *CXCR5* guides Tfh cells into GCs of lymphoid follicles, where they undergo maturation with the assistance of B cells [37,57,58]. The development of GC B cells and plasma cells further depends on the *IL-21* and CD40L signals provided by Tfh cells [19]. Tfh-derived *IL-21* suppresses Tfr cell proliferation [59], and Tfr cells inhibit B-cell-mediated humoral immune responses [60]. However, whether conditional *PKCλ/ι* knockout in CD4^+^ T cells affects Tfh differentiation remains unreported. In this study, we stimulated spleen cells in vitro using PMA and then performed flow cytometry to detect cell types. Our flow cytometry analysis revealed that conditional *PKCλ/ι* deletion in CD4^+^ T cells reduced the proportion of splenic Tfh cells (*CXCR5*^+^PD-1^+^ T cells) among CD4^+^ T cells (Figure 5C,D). While schistosome infection significantly increased Tfh cell proportion in wild-type mice, compared with uninfected knockout mice, infected *PKCλ/ι* knockout mice presented no significant change in the Tfh proportion (Figure 5C,D). Further assessment of *IL-21* secretion capacity revealed significantly elevated *IL-21* expression in Tfh cells from WTSJ mice compared with those from WT controls. In contrast, PKCλ/ι-deficient Tfh cells did not present a significant increase, with no significant difference between the infected and uninfected knockout groups (Figure 5E,F). Although the proportion of cells obtained under PMA stimulation may not fully reflect the physiological state, the core finding of this study lies in revealing the potential differentiation differences in immune cells of *PKCλ/ι* deficiency mice in the stimulation response, which itself has significant immunological implications. RT–PCR analysis confirmed markedly decreased mRNA expression of Tfh-associated genes (*IL-21*, *CXCR5*, and *ICOS*) in the livers of knockout mice (Figure 7C–E). These findings indicate that conditional *PKCλ/ι* deletion in CD4^+^ T cells decreases Tfh cell differentiation and function. Although Tfh cells may originate through multiple pathways [61], reports suggest that Th2 cells are their source in schistosomiasis [62]. Given that Yang et al. [34] demonstrated that *PKCλ/ι* deletion suppresses Th2 differentiation, this could contribute to Tfh downregulation—although the precise mechanisms require further investigation. Critically, the absence of Tfh expansion following infection in *PKCλ/ι* knockout mice indicates that *PKCλ/ι* deficiency constrains schistosomal antigen-driven differentiation of Tfh cells.

Tfr cells are a specialized T-cell subset coexpressing Bcl-6, *CXCR5*, PD-1, and Foxp3 that regulate humoral immunity by suppressing Tfh and B-cell functions, thereby inhibiting T-B-cell interactions [63]. This study revealed that *PKCλ/ι* knockout did not affect *S. japonicum* infection-induced Treg cell differentiation, with no significant difference in Treg levels in the spleen of wild-type and PKCλ/ι-deficient mice (Figure 6A,B). However, the proportion of Tfr cells (Bcl-6^+^Foxp3^+^CD4^+^ T cells) was significantly greater in the spleens of KOSJ mice than in those of WTSJ controls (Figure 6C,D). Bcl-6, a master regulator of both the Tfh and Tfr subsets, is essential for their development [55,64].

Blimp-1 directly binds to enhancers and introns in the 5′ upstream region of *CXCR5*, suppressing *CXCR5* expression in murine Tfr cells and thereby limiting the size of the Tfr population [65]. The results of hepatic RT–PCR revealed that Bcl-6 mRNA levels were significantly higher in KO mice than in WT mice but were lower in KOSJ mice than in WTSJ mice (Figure 7A), whereas Blimp-1 mRNA levels were markedly elevated in PKCλ/ι-deficient mice (KOSJ vs. WTSJ, *p* < 0.01, Figure 7B). These data indicate that *PKCλ/ι* deletion upregulates Blimp-1 in the hepatic tissue of *S. japonicum*-infected mice, inhibiting naïve CD4^+^ T-cell differentiation into the Bcl-6^+^ Tfh/Tfr lineage. However, splenic analysis revealed significantly more Foxp3^+^ Tfr cells within the Bcl-6^+^CD4^+^ T-cell compartment in KOSJ mice than in WTSJ mice (Figure 6C). These findings suggest that under schistosomal antigen stimulation, PKCλ/ι-deficient CD4^+^ T cells preferentially differentiate into Tfr cells, further suppressing Tfh differentiation and B-cell-mediated humoral immunity, but this mechanism requires further investigation.

ScRNA-seq of liver confirmed flow cytometry observations: infected PKCλ/ι-KO (KOSJ) mice exhibited elevated hepatic T-cell proportions (3.45% vs. WTSJ 2.87%) with dramatic expansions in Tregs (36.49% vs. 16.78%) and Tfr cells (30.65% vs. 0.69%), alongside increased total B cells (8.23% vs. 2.96%) and Bregs (12.99% vs. 3.39%) (Figure 8B and Figure 9B, Supplementary Tables S3 and S4). In the spleen tissue, KOSJ mice exhibited attenuated B-cell depletion alongside a significant shift in B-cell function compared to WTSJ mice, with higher proportions of IL-10-producing regulatory B cells. Furthermore, KOSJ mice displayed significantly more follicular regulatory T (Tfr: Bcl-6^+^Foxp3^+^) cells than WTSJ mice. This indicates *PKCλ/ι* deficiency promotes regulatory immune responses during *S. japonicum* infection in spleen. Our integrated analysis demonstrates strong concordance between flow cytometry in spleen and single-cell RNA sequencing (scRNA-seq) in liver in schistosome-induced immune alterations. Of course, the cell proportion data derived from single-cell RNA sequencing should be regarded as an indication of “population-level trends” rather than being used for strict “individual quantitative comparisons”. In future studies, multiple intra-group biological replicate samples should be analyzed to enable biological variability to be captured.

To investigate alterations in cellular communication in CD4^+^ T-cell-specific *PKCλ/ι* knockout mice, we performed single-cell transcriptome sequencing on liver tissues from *S. japonicum*-infected wild-type (WTSJ) and knockout (KOSJ) mice. The results revealed that the proportions of B cells (particularly Bregs) and Treg/Tfr cells were elevated, which is consistent with the findings from the spleen; moreover, the proportion of neutrophils was lower in KOSJ livers than in WTSJ livers. This immune remodeling indicates that *PKCλ/ι* deletion suppresses progranulomatous inflammatory cell subsets [66] while enhancing anti-inflammatory populations. The Th2-type inflammatory response induced by schistosome egg deposition is the core driving force of liver fibrosis [48]. The expansion of immunomodulatory cells likely attenuates fibrogenesis through multiple mechanisms, such as Bregs secreting IL-10/TGF-β to inhibit excessive Th2 polarization and reduce IL-4/IL-13-dependent hepatic stellate cell activation [12]; Tfr cells dampening GC reactions [67], limiting antibody production; and Tregs directly inhibiting Th2 effector functions via IL-10 [68]. The IL-10-driven feedback loop cross-regulation between Bregs and Tregs may sustain immune tolerance [69], this mechanism may explain the synchronous upregulation of multiple types of immunosuppressive cells after the deletion of PKCλ/ι. Furthermore, the decline in the proportion of neutrophils may reduce the secretion of matrix metalloproteinase 9 (MMP-9) [70]. These results demonstrate that *PKCλ/ι* sustains profibrotic Th2 inflammation by repressing Breg/Treg/Tfr differentiation, while its knockout activates a multitiered “IL-10-immunoregulatory network” to counteract fibrosis.

Further sequencing analysis revealed significant differences in intercellular communication between the *S. japonicum*-infected wild-type (WTSJ) and PKCλ/ι-knockout (KOSJ) groups. Compared with WTSJ mice, the KOSJ group presented increased cellular interactions in terms of quantity, diversity, intensity, and complexity. In PKCλ/ι-deficient mice, IL-10 signaling, which is associated with anti-inflammatory responses, was markedly increased, whereas proinflammatory TNF signaling was predominant in infected wild-type mice (Figure 10G).

CellChat interaction analysis confirmed the robust increase in IL-10 pathway activity in KOSJ mice (Figure 11B). Tfr cells primarily mediate immunosuppression via IL-10 signaling to B cells (Figure 11D) via the ligand–receptor pair IL-10–(IL-10ra/IL-10rb). Violin plot analysis of ligand–receptor gene expression revealed that IL-10 was expressed predominantly in Tfr cells, whereas IL-10ra/IL-10rb were expressed in both DC cells and Treg cells (Figure 11F). These findings indicate that *PKCλ/ι* knockout amplifies the intercellular interaction network, with specific enrichment of IL-10 signaling. Further investigation revealed that the strength of the IL-10–IL-10ra/IL-10rb interaction was increased and that the cellular source of IL-10 was dynamically reshaped: IL-10 is primarily secreted by proinflammatory mast cells in wild-type mice but was predominantly produced by Tfr cells in PKCλ/ι-KO mice. RT–PCR analysis revealed significantly higher IL-10 levels in the liver tissues of the KOSJ group than in those of the WTSJ group (Figure 11G). In conjunction with the violin plot (Figure 11F), these findings suggest that IL-10 in hepatic tissues is predominantly secreted by Tfr cells. This observation implies that Tfr cells exert anti-inflammatory and antifibrotic effects through the production of IL-10, a well-characterized anti-inflammatory cytokine. These results align with those of previous studies by TFTT and Chaye et al. [71,72], further supporting the immunomodulatory role of Tfr cells in mitigating liver inflammation and fibrosis. IL-10-secreting B cells are a subset of immunosuppressive cells that play a crucial role in maintaining immune tolerance. These cells exert their immunomodulatory functions primarily through the production of anti-inflammatory mediators, including IL-10, IL-35, and TGF-β [73]. Our findings suggest that *PKCλ/ι* depletion may alter the immune microenvironment by shifting IL-10 secretion from mast cells to Tfr cells. This redistribution likely occurs via a “cell–cell interaction reprogramming” mechanism, highlighting a potential regulatory axis involved in immune suppression.

In the pathological context of schistosomiasis, the upregulation of IL-10 signaling pathway exerts dual protective effects. First, Tfr cell-derived IL-10 directly attenuates hepatic stellate cell activation—triggered by egg deposition—through the suppression of excessive Th2 cell responses and IL-4/IL-13 signaling [74]. Second, IL-10 acts synergistically with Treg and Breg cells to establish an immunosuppressive microenvironment, restricting neutrophil infiltration [75] and thereby mitigating extracellular matrix degradation. Furthermore, an enhanced Tfr–B cell interaction network may amplify the localized immunosuppressive effects of IL-10 via contact-dependent signaling, establishing a self-reinforcing “anti-inflammatory–antifibrotic” feedback loop. This mechanistic framework may explain the marked reduction in liver fibrosis following *PKCλ/ι* knockout. Collectively, our findings suggest a novel therapeutic strategy for schistosomiasis: PKCλ/ι-targeted modulation of IL-10 signaling could represent a precision approach to simultaneously sustain antiparasitic immunity while curbing fibrosis progression.

## 5. Conclusions

This study revealed that conditional knockout of *PKCλ/ι* in CD4^+^ T cells leads to three key immunomodulatory effects: (1) suppression of Tfh cell differentiation, (2) promotion of Tfr cell generation, and (3) a consequent reduction in IL-6 production, the GC reaction, and antibody secretion by effector B cells. Importantly, we demonstrated that increased Tfr cell population and Breg-derived IL-10 secretion collectively contribute to the amelioration of schistosomiasis-associated hepatic fibrosis. These findings establish *PKCλ/ι* as a multifaceted regulator of immune responses during *S. japonicum* infection, offering novel insights into disease pathogenesis and paving the way for the development of targeted therapeutic interventions against schistosomiasis japonica.

## Figures and Tables

**Table 1:** Primer sequence list.

Genes	Sequence (5′-3′)
*18S*	Forward: TGCACCACCAACTGCTTAGC
	Reverse: GTGGTCATGAGCCCTTCCA
*CXCR5*	Forward: ACTCCTTACCACAGTGCACCTT
	Reverse: GGAAACGGGAGGTGAACCA
*IL-21*	Forward: GGAGACTCAGTTCTGGTGGC
	Reverse: GAGCGTCTATAGTGTCCGGC
*ICOS*	Forward: ACCAAGGGAAGCGGAAATG
	Reverse: GGAGCTGTCTGGGTTGTTTAG
*Bcl-6*	Forward: AAAGGCCGGACACCAGTTTT
	Reverse: TCACGGGGAGGTTTAAGTGC
*NF-κB1*	Forward: TCCACTGTCTGCCTCTCTCGTC
	Reverse: GCCTTCAATAGGTCCTTCCTGC
*CD22*	Forward: CCACTCCTCAGGCCAGAAACT
	Reverse: TGCCGATGGTCTCTGGACTG
*FCGR2B*	Forward: GCCTGTCACCATCACTGTCCAAGGGCCCAA
	Reverse: AATGTGGTTCTGGTAATCATGCTCTGTTTCTTC
*LILRB4*	Forward: ATGGGCACAAAAAGAAGGCTAA
	Reverse: GGCATAGGTTACATCCTGGGTC
*Blimp-1*	Forward: GAAGGGAACACGCTTTGGAC
	Reverse: GATTCACGTAGCGCATCCAG

## Data Availability

The original contributions presented in this study are included in the article. Raw RNA-seq reads have been deposited in the NCBI Sequence Read Archive (SRA) under the BioProject accession number PRJNA755071. Individual sample accessions are listed in Supplementary Table S1. Processed gene expression matrices and raw sequencing data are available in the Gene Expression Omnibus (GEO) under accession number GSM9020735 and GSM9020736. The specific information is listed in Supplementary Table S2. Further inquiries can be directed to the corresponding authors.

## Supplementary Materials

The following supporting information can be downloaded at: https://www.mdpi.com/article/10.3390/biom15101430/s1. Figure S1: The gate strategy of flow cytometry; Figure S2: Changes in immune cell proportion in liver by single-cell sequencing; Table S1: Individual sample accessions of RNA-seq; Table S2: Accession number of single-cell sequencing; Table S3: Hepatic immune cell proportions in single-cell sequencing; Table S4: Hepatic T cell subtype proportions in single-cell sequencing.

## Abbreviations

The following abbreviations are used in this manuscript:

Tfr	follicular regulatory T
Breg	regulatory B
BCR	B-cell receptor
aPKC	atypical protein kinase C
SPF	specific pathogen-free
Tfh	T follicular helper
*S. japonicum*	*Schistosoma japonicum*
RT-PCR	Real-time fluorescence quantitative polymerase chain reaction
PKC	Protein kinase C
WT	Wild-type
WTSJ	WT group mice infected with *S. japonicum*
KO	Knockout
KOSJ	KO group mice infected with *S. japonicum*
PMA	Phorbol 12-myristate 13-acetate
