# Supplementary figures and images for: Conditional Ablation of PKCλ/ι in CD4+ T Cells Ameliorates Hepatic Fibrosis in Schistosoma japonicum-Infected Mice via T Follicular Helper (Tfh) Cell Suppression Coupled with Increased Follicular Regulatory T (Tfr) and Regulatory B (Breg) Cell Activities
Source: Biomolecules. 2025 Oct 9;15(10):1430. doi: 10.3390/biom15101430 (PMC12562275; doi:10.3390/biom15101430)

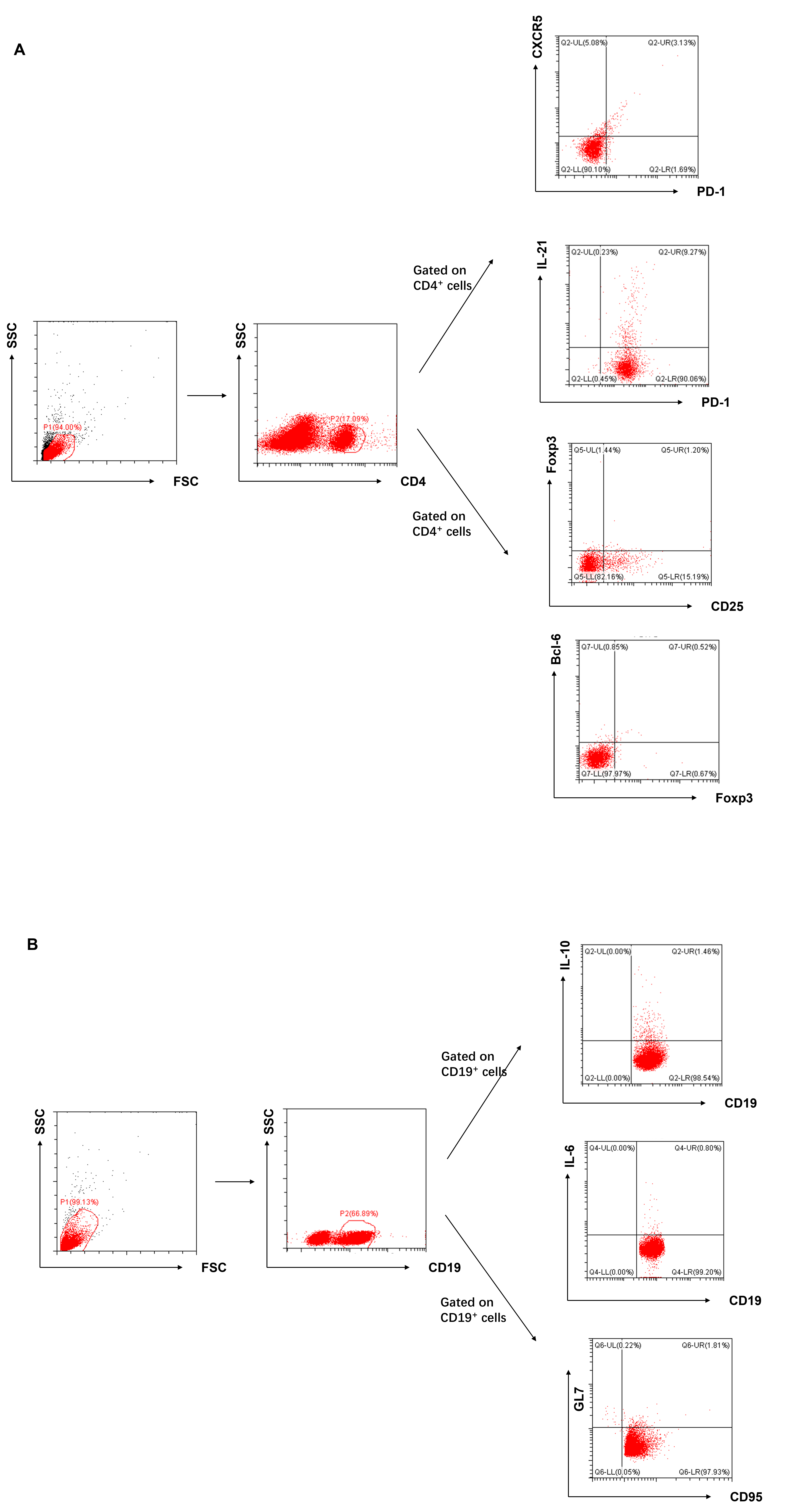

Supplement: Supplementary file 1 [file biomolecules-15-01430-s001.zip › Figure S1.tif]

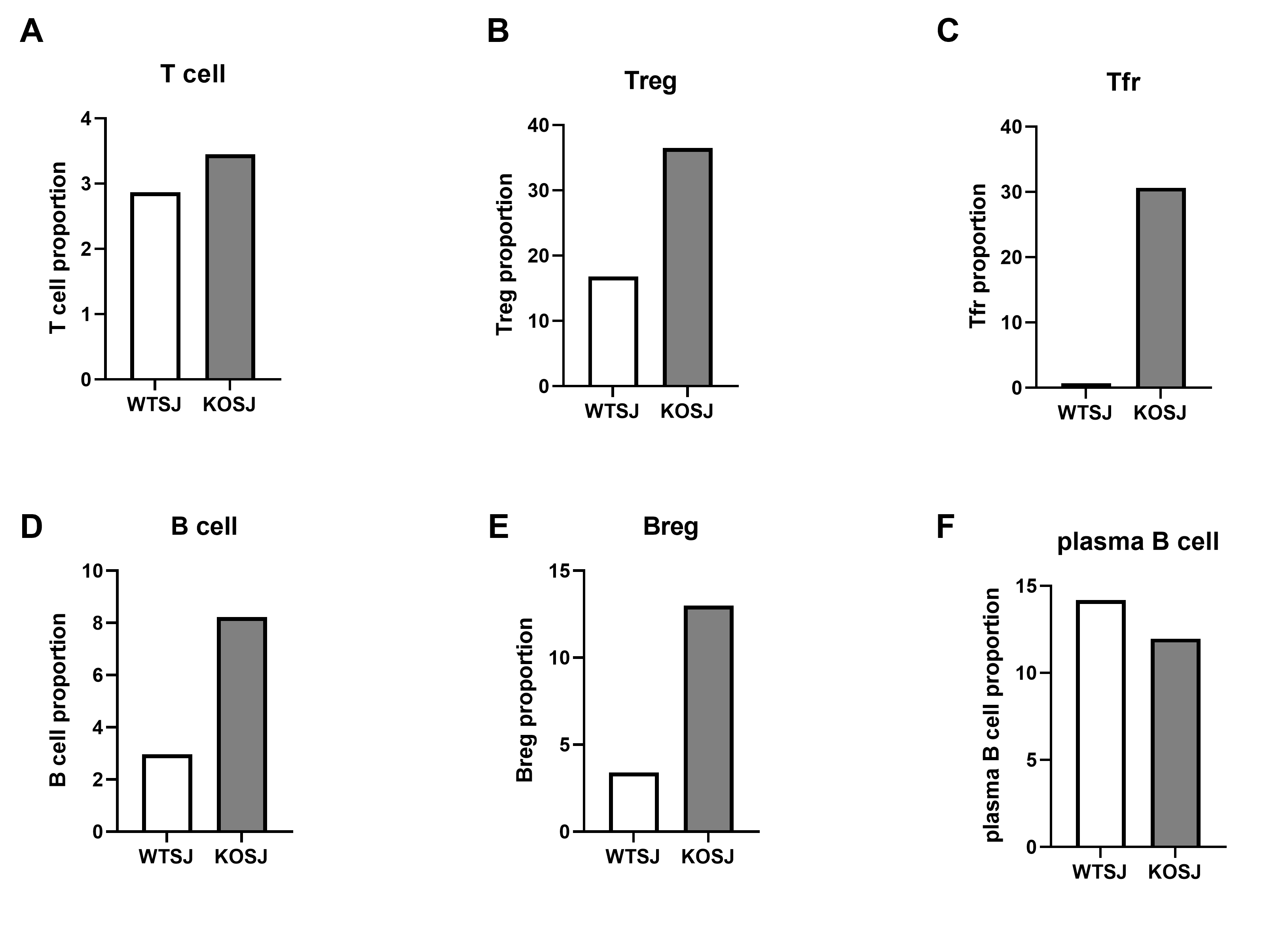

Supplement: Supplementary file 1 [file biomolecules-15-01430-s001.zip › Figure S2.tif]
